# Boron Nitride Nanotube-Based Separator for High-Performance Lithium-Sulfur Batteries

**DOI:** 10.3390/nano12010011

**Published:** 2021-12-21

**Authors:** Hong-Sik Kim, Hui-Ju Kang, Hongjin Lim, Hyun Jin Hwang, Jae-Woo Park, Tae-Gyu Lee, Sung Yong Cho, Se Gyu Jang, Young-Si Jun

**Affiliations:** 1 School of Chemical Engineering, Chonnam National University, 77 Yongbong-ro, Buk-gu, Gwangju 61186, Korea; khs7525169@gmail.com (H.-S.K.); wgguswls@gmail.com (H.J.H.); jaewoopark0218@gmail.com (J.-W.P.); 2 Department of Advanced Chemicals & Engineering, Chonnam National University, 77 Yongbong-ro, Buk-gu, Gwangju 61186, Korea; gmlwn120@gmail.com (H.-J.K.); dlxorb007@gmail.com (T.-G.L.); 3 Functional Composite Materials Research Center, Institute of Advanced Composites Materials, Korea Institute of Science and Technology, Wanju, Jeonbuk 55324, Korea; hongjin3783@gmail.com; 4 Department of Environment and Energy Engineering, Chonnam National University, 77 Yongbong-ro, Buk-gu, Gwangju 61186, Korea

**Keywords:** lithium-sulfur batteries, boron nitride nanotubes, functional separators, lithium-ion transport, shuttle effect

## Abstract

To prevent global warming, ESS development is in progress along with the development of electric vehicles and renewable energy. However, the state-of-the-art technology, i.e., lithium-ion batteries, has reached its limitation, and thus the need for high-performance batteries with improved energy and power density is increasing. Lithium-sulfur batteries (LSBs) are attracting enormous attention because of their high theoretical energy density. However, there are technical barriers to its commercialization such as the formation of dendrites on the anode and the shuttle effect of the cathode. To resolve these issues, a boron nitride nanotube (BNNT)-based separator is developed. The BNNT is physically purified so that the purified BNNT (p−BNNT) has a homogeneous pore structure because of random stacking and partial charge on the surface due to the difference of electronegativity between B and N. Compared to the conventional polypropylene (PP) separator, the p−BNNT loaded PP separator prevents the dendrite formation on the Li metal anode, facilitates the ion transfer through the separator, and alleviates the shuttle effect at the cathode. With these effects, the p−BNNT loaded PP separators enable the LSB cells to achieve a specific capacity of 1429 mAh/g, and long-term stability over 200 cycles.

## 1. Introduction

With recent developments in science and technology, energy demand has increased rapidly, and the demand for secondary batteries has also increased rapidly [1]. Efforts are underway to develop renewable energy generation systems and change internal combustion engines to electric motors in vehicles to replace conventional fossil-based energy due to emergent climate change induced by global warming [2,3]. Renewable energy sources such as solar and wind energy generators require batteries for energy storage systems that can hold excess energy when supply is high for later use [4,5]. Currently, lithium-ion batteries (LIBs) are used for these applications. However, with energy densities of approximately 240–420 Wh/kg, LIBs are reaching their theoretical limits [6,7]. Next-generation batteries must be developed to meet the desire for batteries with higher energy densities and longer lifespans [8].

As an alternative to LIBs, lithium-sulfur batteries (LSBs) have attracted considerable attention [9,10]. The theoretical energy density of lithium-sulfur batteries is approximately 2600 Wh/kg and sulfur, which is used as a cathode in LSBs, is inexpensive and widely abundant [11]. However, there are several problems that hinder the commercialization of LSBs [12]. First, short circuits caused by the formation of lithium metal dendrite shorten the cycle stability [13,14]. Second, the shuttle effect reduces the capacity of LSBs. The shuttle effect occurs when lithium polysulfide (Li_2_S_n_, 4 ≤ *n* ≤ 8) generated during charging and discharging is dissolved in the electrolyte, passes through the separator, and reacts on the surface of the anode. The lithium polysulfide reduces sulfur, lowering capacity and cycle stability [15]. To mitigate these problems, many studies have reported the development of separators in LSBs since it affects the anode and cathode simultaneously. First, carbon-based materials are added to the separator [16]. Carbon-based materials, including graphene, carbon nanotubes, and porous materials, were shown to adsorb the lithium polysulfide to prevent the shuttle effect, and reactivate the eluted polysulfide. However, the interaction with polysulfide was weak, additional functionalization needs to reduce the shuttle effect. Various studies have reported the use of a functionalized polymer separator to alleviate the shuttle effect through attraction or repulsion of lithium polysulfide [17]. However, polymer separators have exhibited low thermal and chemical stability, limiting their practical applications. Finally, metal-oxide-modified separators have been reported to increase the absorption of the polysulfide and increase the thermal and chemical stability of the battery [18,19]. However, these metal-oxide materials exhibit low flexibility.

Boron nitride nanotubes (BNNTs) in which boron and nitrogen are bonded alternately exist in the form of a tube connected by a hexagonal honeycomb structure, similar to carbon nanotubes (CNTs) [20]. Owing to their structural composition, BNNTs have excellent mechanical properties, thermal conductivity, and thermal expansion properties [21]. In addition, since BNNTs possess the characteristics of ceramics, such as chemical and thermal stability in addition to electrical insulation, they are suitable for use as a separator [22]. BNNT separators were reported to increase lithium-ion conductivity due to their wettability for the organic electrolyte. It was reported that B atoms in BNNT interact with the anion of lithium salt by Lewis acid–base interaction improve lithium-ion transfer [23,24]. In addition, BN materials separator, such as hexagonal boron nitride (h−BN) separator, was also reported to increase the stability of the lithium metal anode in lithium-metal batteries owing to their high thermal conductivity [25]. The h−BN separator was reported to have a partial charge on the surface caused by the electronegativity of B and N, which is expected to effectively suppress the elution of lithium polysulfide, thereby reducing the shuttle effect [26,27]. BNNTs, which have the same B–N bonding with h−BN, are expected to alleviate the shuttle effect. However, it is important to note that BNNTs synthesized in large quantities often contain many impurities, and hence, it is necessary to purify BNNTs before using them in LSBs [28,29].

Herein, we investigated BNNT-based separators in LSBs for the first time. The electrochemical behavior of the lithium-sulfur batteries with BNNTs was compared with that of the batteries without BNNTs, batteries using unpurified BNNTs, and batteries using purified BNNTs. Galvanostatic cycling with the potential limit and electrochemical impedance spectroscopy (EIS) were used to analyze the electrochemical behavior of the batteries. The battery cell components were analyzed by scanning electron microscopy (SEM), X-ray photoelectron spectroscopy (XPS), and UV-visible spectroscopy (UV-vis) to understand the mechanism of improved performance of the LSBs with BNNTs.

## 2. Materials and Methods

### 2.1. Materials

Boron nitride nanotubes (BNNTs) were synthesized and kindly provided by Jeonbuk National University High-Enthalphy Plasma Research Center (high-temperature RF-plasma equipment, Wanju, Korea). BNNTs were used after purification to remove impurities and prepared as fluffy powders. Polypropylene separator (PP, Celgard 2400, Celgard Co., Charlotte, NC, USA), polyvinylidene fluoride (PVDF, MTI Co., Richmond, CA, USA), and N-methyl pyrrolidone (NMP, 99.0%, DAEJUNG Co., Siheung, Korea) were used as purchased.

### 2.2. Purification of BNNTs

BNNTs were calcined at 650 °C for 6 h in a box furnace. After calcination, BNNTs were added to deionized water (DI water) and mixed for 30 min at 2500 rpm using a high shear mixer (L5M-A, Young-jin Co., Gunpo, Korea). After mixing, the liquid was left undisturbed, settled down for 1 h to obtain BNNT sediments. The supernatant was then carefully decanted. This process was repeated until the supernatant became transparent. BNNTs were obtained via freeze-drying in tert-butyl alcohol and DI water (4:6; *v*/*v*) for 3 days.

### 2.3. Fabrication of BNNT (or p−BNNT) Loaded Separator

Separators were fabricated using raw BNNT (BNNT) and purified BNNT (p−BNNT), respectively. BNNT (40 mg) and PVDF (10 mg) were dispersed in NMP (2 mL). The mixture was sonicated for 1 h and stirred overnight to obtain a homogeneous slurry. The slurry was cast on a PP separator and dried in a vacuum oven at 50 °C for 24 h. The loading mass of BNNT on the PP separator (x in the BNNT−PP−x) was controlled according to the amount of slurry for drop-casting. p−BNNT loaded separators were also fabricated in an identical manner.

### 2.4. Cell Assembly

The electrolyte was prepared by dissolving 1 M lithium bis(trifluoromethane-sulfonyl)imide (LiTFSI, Sigma-Aldrich Co., Darmstadt, Germany) and 1 wt% lithium nitrate (LiNO_3_, 99.99%, Sigma-Aldrich Co., Darmstadt, Germany) in 1,3-dioxolane (DOL, 99.8% of Sigma-Aldrich Co. Darmstadt, Germany) and 1,2-dimethoxyethane (DME, 99.5%, Sigma-Aldrich Co., Darmstadt, Germany) (1:1 volume ratio). Catholyte, which means cathode material (Li_2_S_6_) with electrolyte, is prepared by mixing sulfur (more than 99.5% in Sigma-Aldrich, Darmstadt, Germany) and Li_2_S (99.9%, Alfa-Aesar, Haverhill, MA, USA) in an electrolyte and heating catholyte at 50 °C. In the diffusion test, catholyte is prepared without LiNO_3_. All batteries were assembled in a glove box filled with high purity argon gas, controlling O_2_ and H_2_O concentration to zero. A 2032-coin cell model was used, and catholyte was prepared by casting 2 mg sulfur per area on carbon cloth (CH900-20, Kuraray Co., Tokyo, Japan) used as a current collector. Anode was lithium metal (0.75 mm thickness, Sigma Aldrich Co., Darmstadt, Germany). For cells containing separators with BNNT, the BNNT faced the cathode.

### 2.5. Characterization

The transmittance of the supernatant in each purification cycle was measured by turbiscan (Linon Tech Co., Hanam, Korea). Morphologies of top views were obtained using scanning electron microscopy (SEM, FEI Nova nano SEM 450) in Korea Institute of Science and Technology (Wanju, Korea). Because BNNTs are non-conductor, the charging phenomenon was very severe during SEM analysis. So, the Pt was coated thicker than the general case, and kV was changed according to the situation. Impurities removal was confirmed using X-ray photoelectron spectroscopy (XPS, Thermo scientific K-Alpha) spectra and X-ray diffraction (XRD, X’pert PRO MRD) measurement in Korea Institute of Science and Technology (Wanju, Korea). In the case of XPS analysis, BNNTs are a non-conductor and have a large peak shift. Therefore, a flood gun was used to reduce the peak shift as much as possible. Diffusion test was quantified using UV-visible spectroscopy (UV-vis, Jasco V-670) in Korea Institute of Science and Technology (Wanju, Korea)

The cycle stability is measured in the potential range between 1.8 and 2.6 V vs. Li/Li^+^ using WBCS 3000 multichannel cycler (WonATech, Seoul, Korea). Electrochemical impedance spectroscopy (EIS) is performed by VSP potentiostat (Bio-Logic, Seyssinet-Pariset, France).

## 3. Results and Discussion

**Figure 1:**
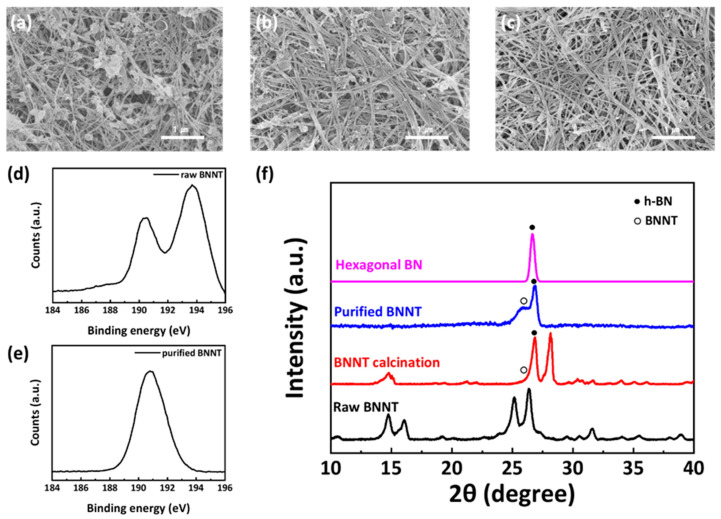
SEM images of (**a**) 1st sediments, (**b**) 8th sediments, (**c**) 12th sediments; XPS B 1 s spectra of BNNT (**d**) before purification and (**e**) after purification; (**f**) XRD patterns of BNNT in the purification process.

Provided boron nitride nanotubes (BNNTs) contain impurities such as amorphous boron, hexagonal boron nitride (h−BN), and amorphous boron nitride (BN) particles [29]. The purification method of BNNT was referred to in previously published papers [30]. The detailed purification process is described in the supplementary information. The scanning electron microscopy (SEM) images of the sediments show that the particles are removed, and the purified BNNTs were obtained (Figure 1a–c). X-ray photoelectron spectroscopy (XPS) spectra and X-ray diffraction (XRD) patterns were analyzed to confirm that the BNNTs were purified. In the XPS spectra, the ratio of boron to nitrogen is about 3 at B 1s peak in XPS spectra. However, after purification, the ratio of boron to nitrogen became 1.2:1, and the high-resolution B 1s XPS of p−BNNT shows only the peak of B–N binding at 191 eV (Figure 1d,e). In the XRD graph, all the amorphous boron peaks have disappeared (Figure 1f). In the XRD graph, all the amorphous boron peaks disappeared after purification. It is important to compare the XRD patterns of BNNT after calcination and purification. One crystalline impurity in BNNT is h−BN, which has a sharp peak at 26.7°. BNNT, which is not crystalline, has a broad peak at 25.8°. The BNNTs after calcination contain a large amount of h−BN, as seen in the strong h−BN peak at 26.7°. After calcination, the broad peak at 25.8° is not evident because of the intensity of the h−BN peak. However, after purification, the h−BN peak is significantly reduced such that the BNNT peak is clearly observed. This indicates the purification method effectively removes particle impurities such as h−BN.

**Figure 2:**
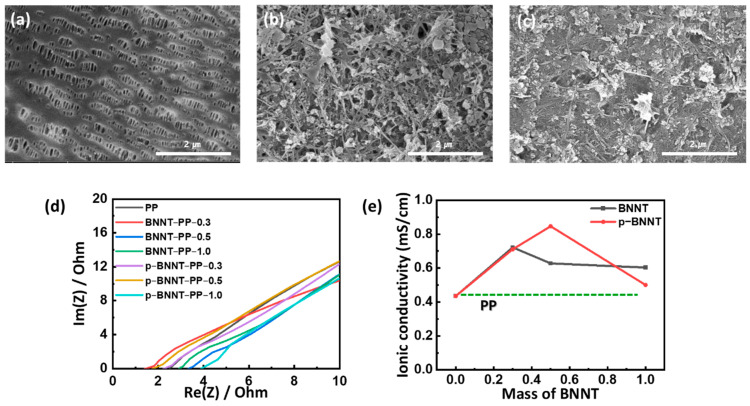
SEM images of (**a**) PP separator (Celgard 2400), (**b**) BNNT separator, and (**c**) p−BNNT separator; (**d**) EIS of separators with Al symmetric electrode cell; (**e**) ionic conductivity of separators.

To confirm the morphology of the BNNT separators, top-views of the polypropylene (PP) PP separator, the BNNT separator, and the p−BNNT separator are observed through SEM. The PP separator is observed to have uniform pores (Figure 2a). It is observed that BNNTs covered the PP separator (Figure 2b,c). 1−dimensional (1D) structure materials are usually stacked randomly to form a 3−dimension spidernet-like structure [31].

Additional materials such as h−BN and boric acid were also coated on the separator, which may interfere with the lithium-ion transport. To evaluate this, lithium-ion conductivity was evaluated using electrochemical impedance spectroscopy (EIS), which measures the resistance to the flow of current. The resistance of each part of the cell was calculated through Nyquist modeling. The bulk resistance measured at the highest frequency measures the resistance of the electrodes, electrolyte, and separator (Figure 2d). To minimize the effects of electrodes and electrolytes, the cathode and anode are composed with Al foil except for sulfur. Impedance was measured according to the mass of BNNT cast on the separator, and the ionic conductivity (*σ*) was obtained through the following equation:

(1) $$\sigma =\frac{d}{R_{b}A}$$

where *d* denotes the thickness, *R_b_* is the bulk resistance, and *A* is the area of the separator. The PP separator showed the lowest value 0.43 mS/cm. In BNNT separators, BNNT−PP−0.3 showed the highest ionic conductivity value 0.71 mS/cm, and it gradually decreased as more mass cast. In p−BNNT separators, BNNT−PP−0.5 showed the highest value 0.84 mS/cm and is still higher than that of PP at p−BNNT−PP−1.0 (Figure 2e). It is confirmed that both BNNTs and p−BNNTs increase ionic conductivity despite pore exclusion through the coating on PP. However, the ionic conductivity of p−BNNT−PPs is lowered when the separator becomes thicker, which indicates that the effect of pore exclusion.

The lithium-ion diffusion coefficient is also measured. It is obtained by assembling lithium-sulfur batteries (LSBs) with BNNT−PP−1.0 and p−BNNT−PP−1.0 that maintain higher ionic conductivity than PP. To obtain the Li-ion diffusion coefficient (*D*_*Li*+_), the results of the EIS spectroscopy analysis were substituted into the following equation:

(2) $$Z^{\prime }=\kappa +\sigma \omega^{-0.5}$$

(3) $$D_{Li^{+}}=\frac{R^{2}T^{2}}{2A^{2}N^{4}F^{4}C^{2}\sigma^{2}}$$

*Z*′ is the real part of the resistance, *ω* is the angular frequency, *R* is the gas constant, *T* is the absolute temperature, *A* is the area of the electrode, *N* is the number of electrons transferred in the redox reaction, *F* is the Faraday constant, and *C* is the concentration of lithium-ions in moles. The *σ* can be obtained from the slope of a linear plot of the Warburg resistance versus the angular frequency. The BNNT separator showed the lowest diffusion coefficient of 16.3 × 10^−19^ cm^2^/s and the p−BNNT separator showed the highest diffusion coefficient of 25.7 × 10^−19^ cm^2^/s, which implied that the lithium-ion diffusion coefficient is enhanced when using the p−BNNT separator in the LSBs (Figure S3).

**Figure 3:**
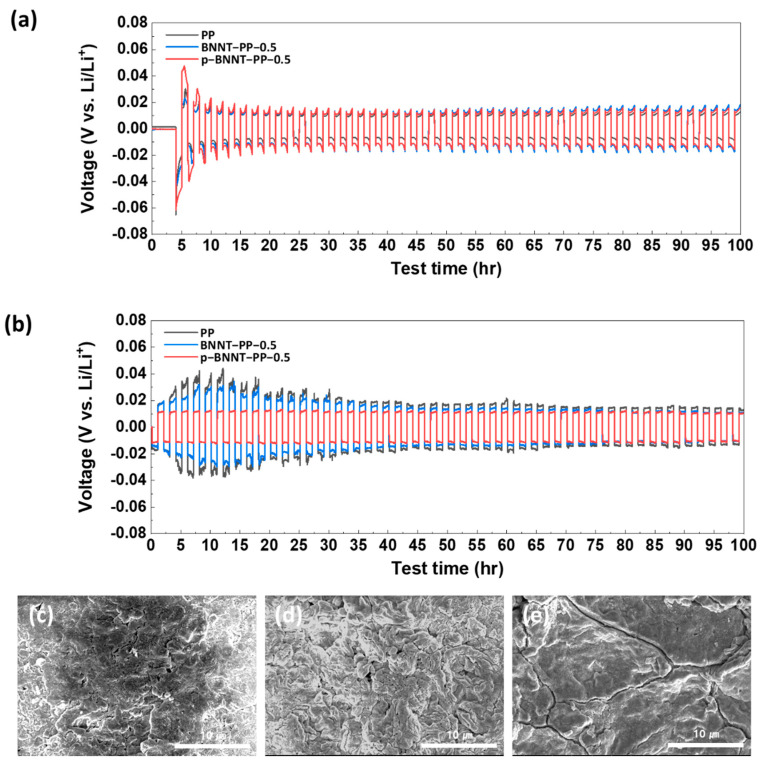
Lithium stripping/plating experiments with PP, BNNT, and p−BNNT loaded separator at (**a**) 0.35 mA/cm^2^ and (**b**) 1 mA/cm^2^; SEM images of lithium metal after finishing lithium stripping/plating measurement using (**c**) PP, (**d**) BNNT−PP−0.5, and (**e**) p−BNNT−PP−0.5.

Lithium stripping and plating analysis were conducted to evaluate the stability of lithium metal by measuring the overpotential that occurred when repeatedly stripping and plating lithium under constant current. To isolate the effect of the separator, a symmetric cell was composed of lithium metal and without sulfur. When stripping/plating was performed at 0.35 mA/cm^2^, a similar overpotential was measured in all separators. However, when stripping/plating was conducted at 1 mA/cm^2^, the overpotentials of the PP separator and the BNNT separator rapidly increased before decreasing and stabilizing (Figure 3a,b). This occurs when dendrites form and detach due to non-uniform lithium stripping/plating. The PP separator had a higher overpotential than that of the p−BNNT separator, and the higher ionic conductivity of the p−BNNT separator increased the stability of the lithium-metal anode (Figure 3c–e). Lithium stripping and plating analysis were also conducted with BNNT−PP−1.0 and p−BNNT−PP−1.0 at 1 mA/cm^2^. The overpotential of p−BNNT−PP−1.0 is slightly lower than that of BNNT−PP−1.0 (Figure S4).

**Figure 4:**
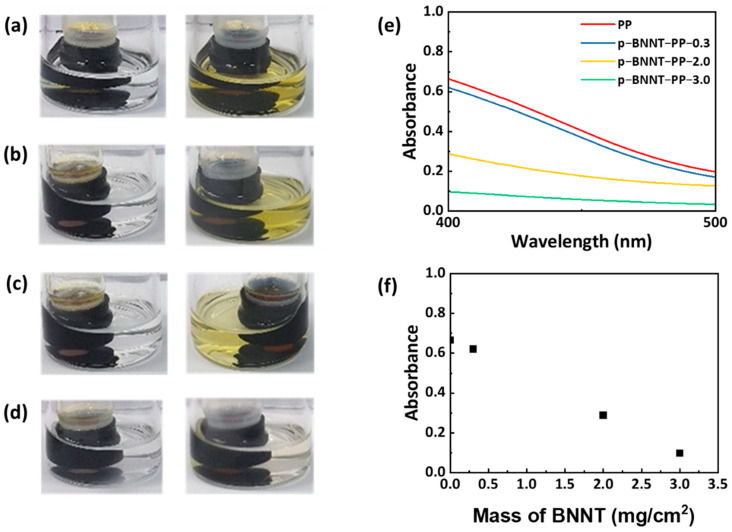
Diffusion test during 6 h with (**a**) PP, (**b**) p−BNNT−PP−0.3, (**c**) p−BNNT−PP−2.0, and (**d**) p−BNNT−PP−3.0 for 6 h from left to right; (**e**) UV-vis spectra of diffused solution; (**f**) quantification graph of UV-vis spectra at 400 nm.

Adsorption tests were conducted to analyze the polysulfide adsorption capacity of BNNTs. First, to check the adsorption capacity of the powder itself, each of BNNT or p−BNNT (20 mg) was added to 4 mL of catholyte diluted with 5 mM and stirred overnight. However, no visible color change was observed even after stirring (Figure S5a,b). That is, BNNT and p−BNNT do not interact strongly with polysulfide. Ex-situ XPS analysis was performed to confirm that the interaction. Porous carbon cloth (CC), which is the current collector, has no affinity with polysulfide. BNNTs on CC and p−BNNTs on CC were analyzed. At the S 2p peak of XPS spectra from porous carbon cloth, the only peak of polysulfide is observed. On the other hand, at the S 2p peak of XPS spectra from BNNT and p−BNNT, a secondary peak appeared, which means interaction with polysulfide and BNNT or p−BNNT. (Figure S5c). To visualize the blocking ability of p−BNNT separator, a diffusion test of lithium polysulfide was conducted. A separator is placed in an open cap vial, and catholyte is added there. A blank electrolyte was added to the outer vial to form a concentration gradient. At this time, PP separator and p−BNNT separator are prepared at 0.3, 2, and 3 mg/cm^2^, and the degree of diffusion is observed after 6 h. It is observed that as the amount of p−BNNT increased, the amount of polysulfide diffusion decreased (Figure 4a–d). To measure diffusion quantitatively, absorbance is measured using UV-visible spectroscopy (UV-vis). At 400 nm peak, it is confirmed that the amount of p−BNNT was reduced by 85% compared to the PP separator when the amount was 3 mg/cm^2^ (Figure 4d,e). It is confirmed that the p−BNNT separators could alleviate the shuttle effect.

**Figure 5:**
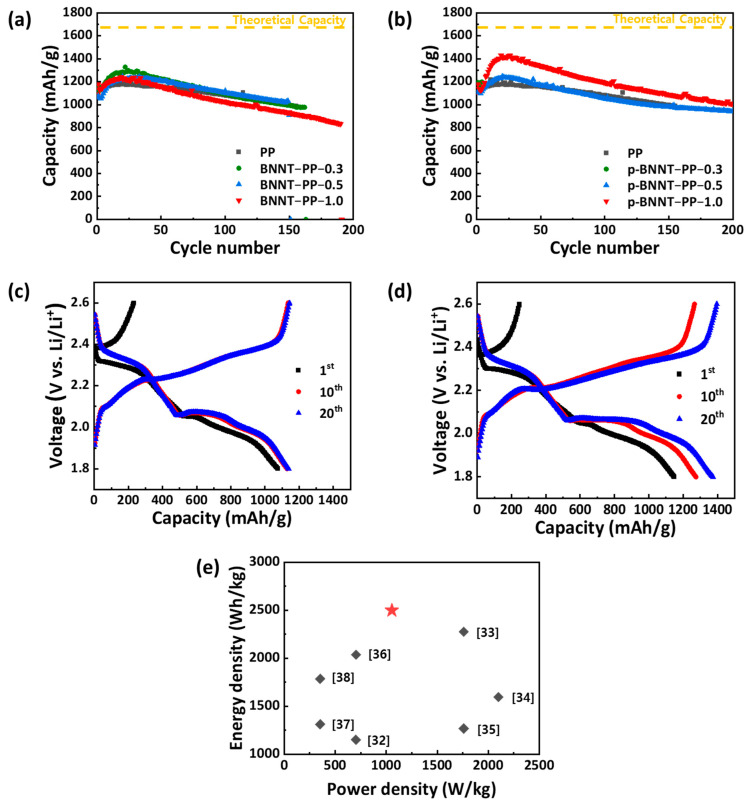
Cycle stability of LSBs with (**a**) BNNT−PP separators, (**b**) p−BNNT−PP separators; Charge/Discharge profile of LSBs of (**c**) PP and (**d**) p−BNNT−PP−1.0 separator at 1st, 10th, 20th cycle. Sulfur loading mass is 2 mgS/cm^2^ and working voltage is 1.8–2.6 V vs. Li/Li^+^ at 0.3 C-rate; (**e**) Ragone plot of LSBs equipped with functional separators so far reported including p−BNNT−PP−1.0 at 100th cycles. These values solely consider the mass of active materials.

After confirming the effect of the BNNT and p−BNNT separators on each component of the cell, the electrochemical performances of the LSBs were measured. First, galvanostatic cycling was performed. Most separators showed no significant difference from the PP separator, but the p−BNNT−PP−1.0 showed a considerably higher capacity. The PP separator showed a discharge capacity of 1197 mAh/g, and the p−BNNT−PP−1.0 showed a discharge capacity of 1429 mAh/g after activation. Considering that the theoretical capacity of sulfur was 1675 mAh/g, the sulfur utilization in the PP separator was 72.6%, and the sulfur utilization in the p−BNNT−PP−1.0 was 85.3%. For the PP separator, the cell no longer worked after 155 cycles, but the p−BNNT−PP−1.0 worked for more than 200 cycles (Figure 5a,b). This capacity and stability improvement was due to the higher lithium ionic conductivity, diffusivity, and stabilization of lithium metal when using the p−BNNT separator. In the discharge profile of the p−BNNT separator, the capacity from 2nd plateau, which means the conversion of the low polysulfide (Li_2_S_n_; 1 ≤ *n* ≤ 4) became more dominant because the blocked polysulfide contributed to the capacity (Figure 5c,d). A comparison of the energy density at 100th cycle with those of other separators utilized in LSBs is presented in Figure 5e. Such a high energy density is due to the high retention of specific capacity at 100th cycle when using p−BNNT−PP−1.0 as a separator [32,33,34,35,36,37,38].

## 4. Conclusions

In conclusion, it is confirmed that the effect of boron nitride nanotube-based separator in lithium-sulfur batteries at the first time. p−BNNT, 1−dimensional structure materials, are stacked randomly to form a 3−dimensional spidernet-like structure on PP separator. Despite of pore-excluding morphology, the ionic conductivity of p−BNNT separator is 0.50 mS/cm, which is higher than that of PP. The diffusion coefficient of p−BNNT separator shows the highest value 25.7 × 10^−19^ cm^2^/s. In addition, lithium metal anode is stabilized by using p−BNNT separator. Via adsorption test and diffusion test, it is confirmed that BNNT separators alleviate shuttle effect by pore exclusion and interaction with polysulfide. Through these advantages, boron nitride nanotube-based separator, especially p−BNNT−PP−1.0, shows higher energy density (1429 mAh/g) and cycle stability (over 200 cycles) than conventional PP separator. The LSB cells with p−BNNT−PP−1.0 show superior performance in terms of gravimetric energy density and power density as compared to those of other LSBs with separators based on carbons, polymers, and metal oxides.

## Data Availability

The data that support the plots within this paper and other findings of this study are available from the corresponding authors on reasonable request.

## Supplementary Materials

The following are available online at https://www.mdpi.com/article/10.3390/nano12010011/s1, Figure S1: BNNTs photo images; Figure S2: Photo images, transmittance, and SEM images of supernatant by cycle; Figure S3: EIS spectras and EIS spectra linear regressions; Figure S4: Lithium stripping/plating experiments with BNNT−PP−1.0 and p−BNNT−PP−1.0 at 1 mA/cm^2^; Figure S5: Photo images of BNNT powder in catholyte and XPS S 2p spectra.
