# Supplementary material for: Boron Nitride Nanotube-Based Separator for High-Performance Lithium-Sulfur Batteries
Source: Nanomaterials (Basel). 2021 Dec 21;12(1):11. doi: 10.3390/nano12010011 (PMC8746311; doi:10.3390/nano12010011)
Supplement: Supplementary file 1 [file nanomaterials-12-00011-s001.zip › nanomaterials-1503588-SI-after.pdf]

nanomaterials

Boron Nitride Nanotube-Based Separator for High-Performance Lithium-Sulfur Batteries

Hong-Sik Kim [1], Hui-Ju Kang [2], Hongjin Lim [3], Hyun Jin Hwang [1], Jae-Woo Park [1], Tae-Gyu Lee [2], Sung Yong Cho [4,*], Se Gyu Jang [3,*] and Young-Si Jun [1,2,*]

1 School of Chemical Engineering, Chonnam National University, 77 Yongbong-ro, Buk-gu, Gwangju 61186, Korea; khs7525169@gmail.com (H.-S.K.); wgguswls@gmail.com (H.J.H.); jaewoopark0218@gmail.com (J.-W.P.)
2 Department of Advanced Chemicals & Engineering, Chonnam National University, 77 Yongbong-ro, Buk-gu, Gwangju 61186, Korea; gmlwn120@gmail.com (H.-J.K.); dlxorb007@gmail.com (T.-G.L.)
3 Functional Composite Materials Research Center, Institute of Advanced Composites Materials, Korea Institute of Science and Technology, Wanju, Jeonbuk 55324, Korea; hongjin3783@gmail.com (H.L.)
4 Department of Environment and Energy Engineering, Chonnam National University, 77 Yongbong-ro, Buk-gu, Gwangju 61186, Korea
* Correspondence: syc@jnu.ac.kr (S.Y.C.), segyu.jang@kist.re.kr (S.G.J.), ysjun@jnu.ac.kr (Y.-S.J.); Tel.: +82-(63)-530-1862 (S.Y.C.); +82-(63)-219-8167 (S.G.J.); +82-(62)-530-1812 (Y.-S.J.)

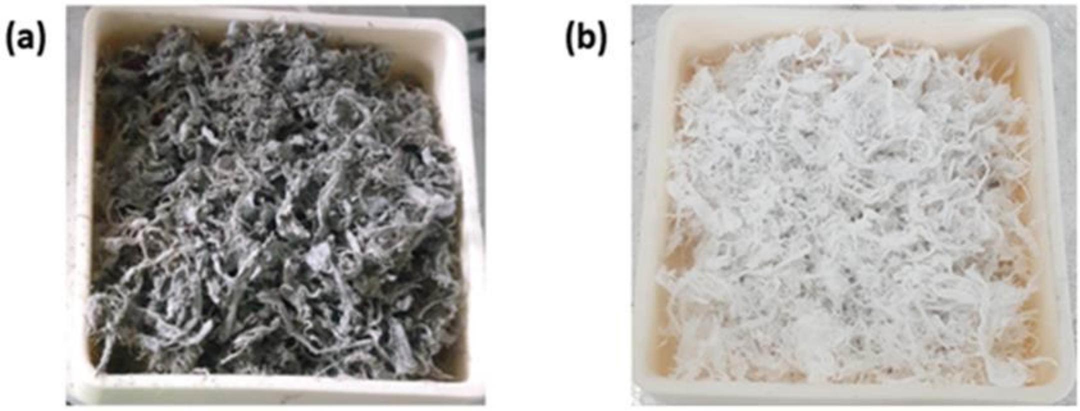

Figure S1. BNNTs photo image (**a**) raw BNNT, (**b**) calcined BNNT.

Boron nitride nanotubes (BNNTs) purification process is as follows. First, to remove amorphous boron, it is calcined at 650 °C for 6 hours to form boron oxide. Color of BNNTs is originally white, but in the case of synthesized BNNTs, they have dark gray color due to the amorphous boron particles it contains. Amorphous boron becomes boron oxide, which increases the weight, and the color changes to white as well (Figure S1). Next purification step was performed in DI water using the dispersibility of the solvent. Using a high shear mixer, a strong shear was applied to the BNNTs in the DI water. BNNTs and impurities are separated from each other by shear force. When mixing is stopped and settled down, nanotubes form bundles and sink due to poor dispersibility. On the other hand, relatively small particles compared to the bundle like h−BN remain in the supernatant. By removing supernatant repeatedly, impurities are removed mechanically. At this time, the purity of BNNT can be confirmed by analyzing the obtained supernatant and sediment.

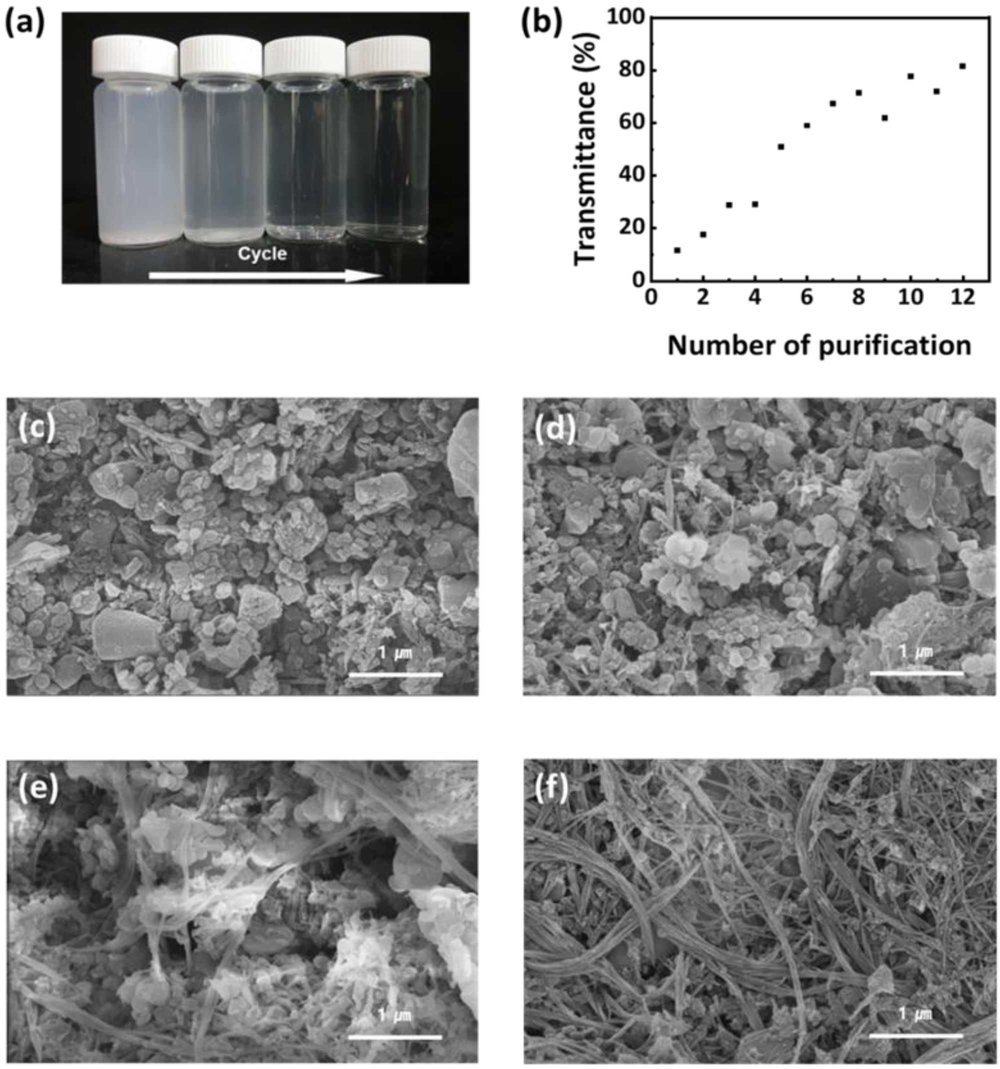

Figure S2. (**a**) Photo images of supernatant 1st, 4th, 8th, 12th in order; (**b**) Transmittance of supernatant; SEM image of supernatant by cycle; (**c**) 1st, (**d**) 4th, (**e**) 8th, (**f**) 12th.

The transmittance of Supernatant was confirmed through turbiscan. It was confirmed that the supernatant became more and more transparent even with a simple optical image (Figure S2a). When the supernatant is measured with turbiscan, the transmittance was saturated at 80% in 12 times (Figure S2b). Even if BNNTs formed bundles, the purification process is stopped when the transmittance is saturated because debundled BNNTs are also present in the supernatant. The SEM image of supernatant showed that the impurities were separated. SEM images of 1st and 4th supernatant at the beginning of purification showed that particles, which are impurities of BNNT, were effectively removed (Figure S2c,d). However, as shown in the SEM images of 8th and 12th supernatant, as the purification continues, BNNTs is observed in the supernatant. In the 12th cycle, where the supernatant is almost transparent, a large amount of BNNTs that have not formed a bundle are also removed. (Figure S2e,f)

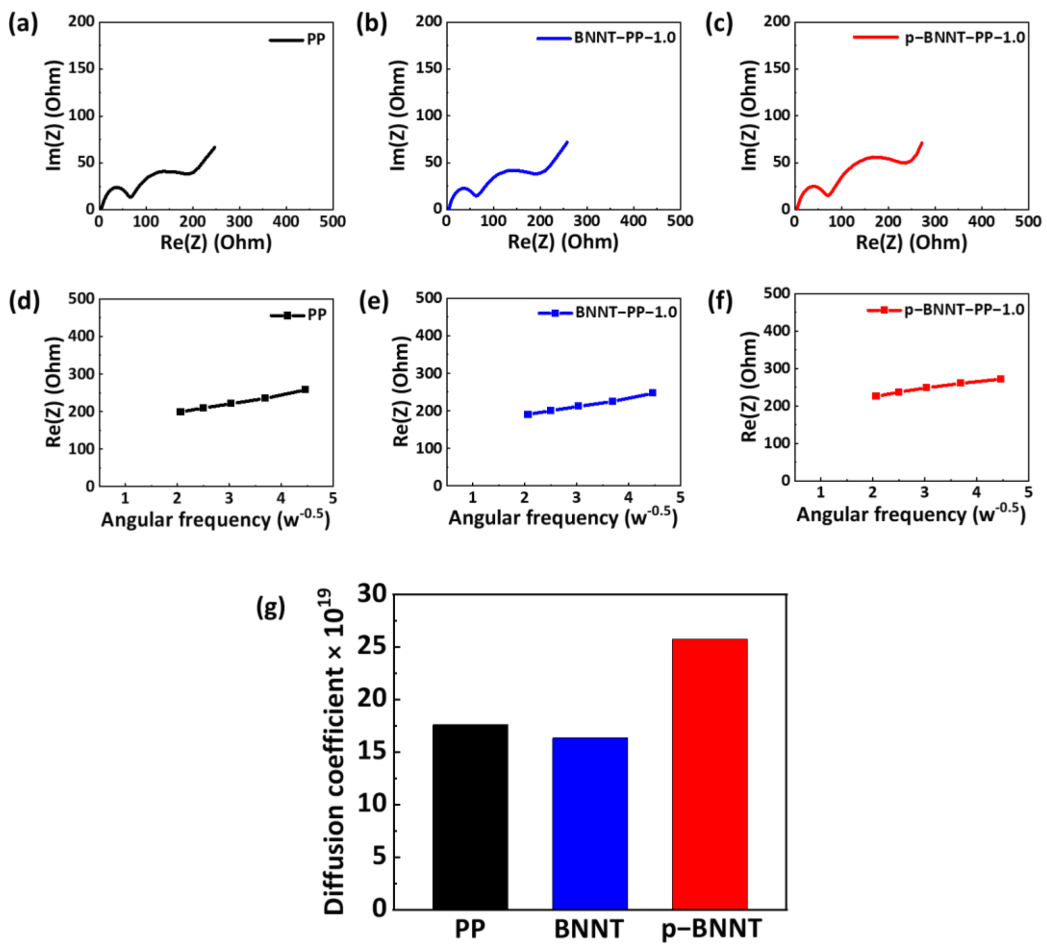

Figure S3. EIS spectra of (**a**) PP, (**b**) BNNT−PP−1.0, (**c**) p−BNNT−PP−1.0; EIS spectra linear regression of (**d**) PP, (**e**) BNNT−PP−1.0, (**f**) p−BNNT−PP−1.0; (**g**) Diffusion coefficient of PP, BNNT−PP−1.0, and p−BNNT−PP−1.0

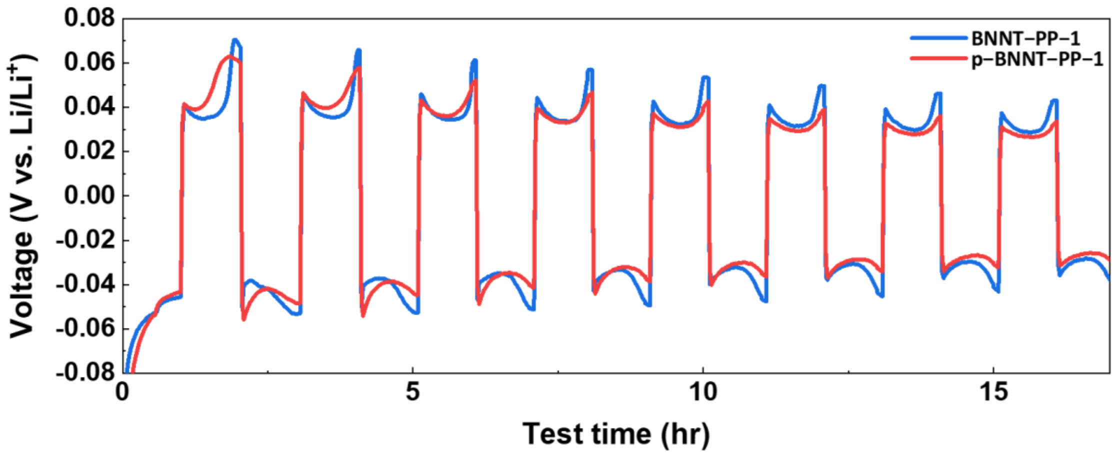

Figure S4. Lithium stripping/plating experiment with BNNT−PP−1.0 and p−BNNT−PP−1.0 at 1 mA/cm^2.

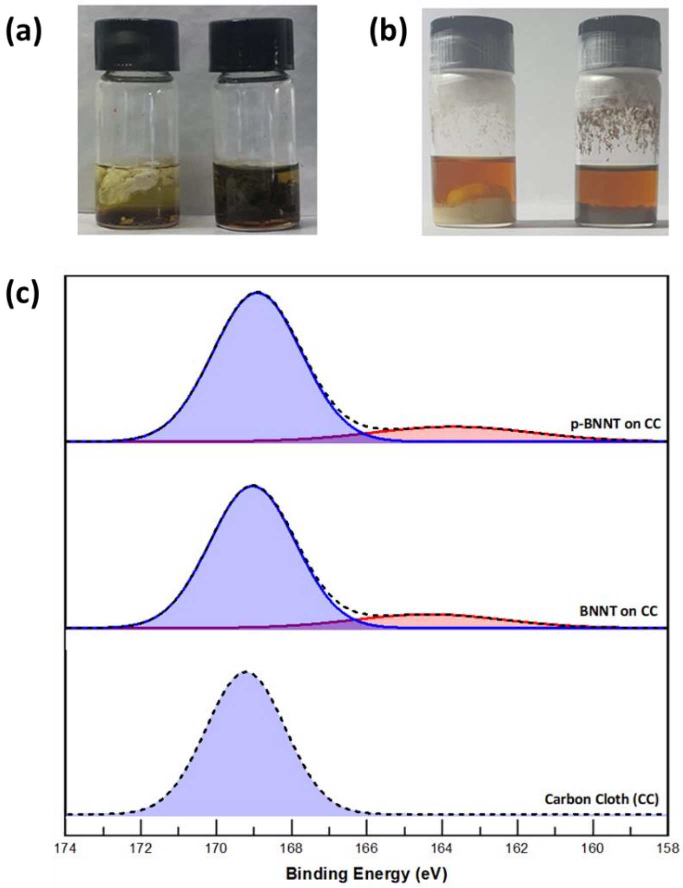

Figure S5. Photo images of BNNTs powder in catholyte (**a**) before adsorption test and (**b**) after adsorption test, through stirring and centrifuging (left side is p−BNNT, right side is BNNT); (**c**) XPS S 2p spectra of adsorption test with Carbon Cloth (CC), BNNT on CC, and p−BNNT on CC.
